# Prognostic Relevance of Expression of *EMP1*, *CASP1*, and *NLRP3* Genes in Pediatric B-Lineage Acute Lymphoblastic Leukemia

**DOI:** 10.3389/fonc.2021.606370

**Published:** 2021-03-05

**Authors:** Jay Singh, Sarita Kumari, Mohit Arora, Deepak Verma, Jayanth Kumar Palanichamy, Rajive Kumar, Gunjan Sharma, Sameer Bakhshi, Deepam Pushpam, M. Shadab Ali, Amar Ranjan, Pranay Tanwar, Shyam S. Chauhan, Archna Singh, Anita Chopra

**Affiliations:** ^1^Laboratory Oncology Unit, Dr. B.R. Ambedkar-Insitute Rotary Cancer Hospital (BRAIRCH), All India Institute of Medical Sciences (AIIMS), New Delhi, India; ^2^Department of Biochemistry, AIIMS, New Delhi, India; ^3^Department of Pathology, Mahavir Cancer Sansthan, Patna, India; ^4^Department of Medical Oncology, AIIMS, New Delhi, India; ^5^Department of Pulmonary Medicine, AIIMS, New Delhi, India

**Keywords:** *EMP1*, *CASP1*, *NLRP3*, B-ALL, leukemia, prednisolone resistance

## Abstract

Glucocorticoid (GC), such as prednisolone, is an essential component of multidrug chemotherapy regimen for pediatric acute lymphoblastic leukemia (ALL). Resistance to GC in leukemia cells is associated with disease progression and poor prognosis. Despite the extensive use of GC for many years, molecular mechanisms underlying its resistance in ALL have not been fully uncovered. Recent studies have shown a potential role of *EMP1*, *CASP1*, and *NLRP3* genes in prednisolone response. In this study on 148 pediatric B-ALL patients, we studied these three genes to assess their association with prednisolone response measured by day 8 blast count after 7 days of induction therapy with prednisolone. Intriguingly, ALL samples exhibited higher expression of *EMP1* along with a low expression of *CASP1* and *NLRP3* compared to disease free normal bone marrow collected from patients with solid tumors. Among the three analyzed genes, only *EMP1* was found to be overexpressed in prednisolone poor responders (p=0.015). Further, a comparison of gene expression between cytogenetic subtypes revealed higher expression of *EMP1* in BCR-ABL subtype. Expression of *EMP1* in multiple gene expression datasets was used for gene set enrichment analysis, which revealed TNF-α, IL-2-STAT5 signaling, inflammatory responses and hypoxia as the major positively associated pathways and E2F targets as negatively associated pathways. Interestingly, the clinical remission rate was higher in *CASP1* high patients (p=0.048). In univariate survival analysis, higher *EMP1* expression was associated with poor prognostic measures while higher expression of *NLRP3* and *CASP1* was associated with better prognostic measures in our data. Further, multivariate analysis revealed an independent association of high *CASP1* and *NLRP3* with a better prognosis. This study strengthens the available evidence that mRNA expression of *EMP1*, *CASP1*, and *NLRP3* may serve as potential biomarkers for risk stratification of pediatric B-ALL patients.

## Introduction

Acute lymphoblastic leukemia (ALL) is the most common childhood malignancy (1). The prognosis of ALL has improved tremendously with the advent of modern combination chemotherapy, amongst which, prednisolone (PRD) serves as an integral component (2, 3). PRD is a glucocorticoid (GC) that exerts its cytotoxic effect on leukemic cells through induction of apoptosis. It has been known over the last 5-6 decades that PRD can induce remission in ALL patients (4). ALL therapy usually involves the administration of systemic PRD for the initial 7-day of treatment. The response to PRD is evaluated by either assessing the absolute number of blasts in the peripheral blood (PB) of the patients on day 8 of induction chemotherapy or by *in vitro* methyl thiazole tetrazolium (MTT) assay on primary culture of leukemic cells collected at the time of diagnosis (2, 3, 5, 6). In the Berlin-Frankfurt-Münster (BFM) protocol, the ALL patients have termed PRD good responders (PGRs) if, at the 8^th^ day after PRD treatment, the blast count <1X10^9^/L and PRD poor responders (PPRs) if the blast count is ≥1X10^9^/L (2, 7). Importantly, poor responders receive intensified therapy, both during the induction and at later phases of the treatment.

The sensitivity of leukemic cells to PRD, as measured by *in vivo* and *in vitro* assays, is also predictive of poor patient outcome in childhood ALL (5, 7–12). Unfortunately, patient response to PRD is highly variable and approximately 10%–15% of pediatric ALL patients, who receive therapy, exhibit poor response to PRD (6). Furthermore, resistance to PRD is observed more frequently in ALL relapse than for other chemotherapeutic agents (5, 13). Therefore, PRD resistance in ALL poses a clinical challenge to oncologists.

In the past decades, extensive studies have been performed to understand the molecular mechanisms underlying GC resistance (14, 15). Paugh et al., in a multi-institutional study (15), evaluated the response of PRD on primary leukemia cells from 444 pediatric ALL patients by genome-wide association study. They found increased expression of key components of the NALP3 pathway - *CASP1* (encoding caspase 1) and its activator *NLRP3* in GC-resistant leukemia cells compared to GC-sensitive leukemia cells. In comparison with the genome-wide analysis of DNA methylation, they found significantly decreased somatic methylation of the promoters of *CASP1* and *NLRP3* genes in GC-resistant ALL cells. They further showed that CASP1 modulates the biological and pharmacological effects of GC by cleaving and inactivating its receptors. In another study, Ariës et al., in a search for druggable targets that could be influencing prednisone resistance, utilized microarray gene expression profile (GEP) in 256 pediatric ALL patients and identified an over 3-fold increase in epithelial membrane protein 1 (*EMP1*) in GC resistant patients (16). Furthermore, *EMP1*-high B-ALL patients showed a significantly worse 5-year event-free survival (EFS) compared with *EMP1*-low patients.

In this study on 148 B-ALL pediatric patients from India, which were treated with ICiCLe protocol (a modified BFM protocol), we aimed to evaluate the association between response to prednisolone of ALL patients, as assessed by day 8 PB blast counts, with mRNA expression levels of *CASP1, NLRP3*, and *EMP1* genes. We also determined the association of expression of these genes with clinical characteristics, cytogenetic findings, post-induction minimal residual disease (MRD), and patient outcome.

## Materials and Methods

In this study, bone marrow (BM) and/or PB samples at the time of diagnosis before initiation of therapy were obtained from pediatric B-ALL patients before the start of the treatment from the Department of Medical Oncology, Dr. BRAIRCH, AIIMS, New Delhi, India. B-ALL cases (n=148) with information on the initial response to prednisolone (measured by day 8 peripheral blood blast count) were included in this study. The diagnosis of B-ALL was based on morphology, cytochemistry, and immunophenotyping. Also, we included 10 BM controls collected from patients with solid tumors where BM examination was done for staging. The study was approved by the institute’s ethics committee and informed consent was obtained from patients and/or their legal guardians in accordance with the Declaration of Helsinki.

### Assessment of Glucocorticoid Response

Initial response to prednisolone (referred to as prednisolone response from hereon) was assessed by counting blasts in the PB smears collected at day 8 of initiation of PRD therapy. The patients were termed PGR (sensitive) and PPR (resistant) based on the presence of day 8 PB blast count of <1 X 10^9^/L and ≥1 X 10^9^/L blasts, respectively.

### Determination of Expression Levels of *CASP1, NLRP3*, and *EMP1* by RQ-PCR

Total RNA was extracted from blast enriched mononuclear cells isolated from BM/PB samples collected at the time of diagnosis using the TRIzol method (ThermoFisher, Scientific, USA). The concentration and quality of RNA were determined with spectrophotometer. RNA was reverse transcribed to cDNA using random hexamers, RNase inhibitor, dNTPs, and M-MuLV reverse transcriptase enzyme (Fermentas, USA). The expression levels of *CASP1, NLRP3* and *EMP1* genes were measured by real-time PCR (CFX96™, Bio-Rad, Hercules, CA, USA) using SYBR Green PCR master mix (Bio-Rad, CA, USA). The primers used were: *CASP1*-F: 5’-GTGCAGGACAACCCAGCTAT-3’ and *CASP1*-R: 5’-TGCGGCTTGACTTGTCCATT-3’. *NLRP3*-F: 5’-GGAGGAGGACTTCGTGCAAA-3’ and *NLRP3*-R: 5’-GAGAAACCCCAGGGACAGTG-3’. *EMP1*-F: 5’-GCCAATGTCTGGTTGGTTTCC-3’ and *EMP1*-R: 5’-GAGGGCATCTTCACTGGCATA-3’.The housekeeping genes used were as per recommendation by a previous study (17): *ABL1* and *PPIA*. In all cases, the samples were run in triplicates. The Ct values were normalized with housekeeping genes. The relative *EMP1*, *CASP1*, and *NLRP3* expression were calculated using the comparative cycle threshold method.

### Assessment of Expression of *EMP1, CASP1*, and *NLRP3* in NCBI-Gene Expression Omnibus Datasets

We downloaded two microarray datasets of GEP of GC-resistant and sensitive ALL patients from NCBI-gene expression omnibus (NCBI‐GEO) (https://www.ncbi.nlm.nih.gov/geo/). The accession numbers of the datasets were GSE5820 (18) and GSE19143 (19). GSE5820 dataset had 13 GC-sensitive and 16 GC-resistant samples whereas the GSE19143 dataset had 14 non-infant GC-sensitive and 13 non-infant GC-resistant samples. In both datasets, the patients were divided based on *in vitro* PRD cytotoxicity which was determined using the MTT assay. In addition, *in vivo* response to PRD was also determined using absolute blast count at day 8 after the 7-day PRD window, in the latter dataset. We compared expression patterns of *EMP1, CASP1*, and *NLRP3* genes between PRD sensitive and resistant patients in both datasets.

### Pathway Analysis

GEP of diagnostic BM from 649 B-ALL patients was extracted from Microarray Innovations In Leukemia (MILE) study data deposited at NCBI-GEO with accession number GSE13159 (https://www.ncbi.nlm.nih.gov/geo/) (20). Similarly, normalized GEP of GSE5820 and GSE19143 was extracted from NCBI-GEO. A correlation (Spearman’s rank correlation coefficient) of expression of all genes in the datasets with *EMP1* was determined using Pingouin statistical package in Python 3 (21). Genes with Spearman’s correlation false detection rate (FDR) corrected p >0.05 were filtered out and remaining genes were arranged in accordance with decreasing Spearman’s r-value, thus creating a ranked correlation file. This file was used as input in the “Preranked GSEA module” of GSEA software from Broad Institute (http://www.broad.mit.edu/gsea) with molecular signature database hallmark gene set (version 7.1) taken as reference gene set (22). Genes set enrichment in the respective pathways were represented as image outputs from GSEA software along with estimated normalized enrichment score (NES), FDR corrected p-value.

### Treatment

All the patients were treated uniformly by Indian Childhood Collaborative Leukemia Group protocol (Unpublished-CTRI/2015/12/006434) which is BFM based protocol (23). All patients were treated with steroid prophase for 7 days. On day 8 PB was examined for blasts and on day 35 BM was examined for remission status and MRD. Patients with age >1 year and age <10 years, PGRs, total leukocyte count (TLC) < 50X10^9^/L, no high-risk cytogenetics, absence of central nervous system disease, and end of induction MRD <10^-3^ were classified as standard risk. Patients with good risk features but age ≥ 10 years or WBC > 50X10^9^/L or bulky lymph nodes/liver/spleen or testicular disease, in absence of other high-risk criteria, were classified as intermediate risk. All PPRs, high-risk cytogenetics, central nervous system disease, and MRD > 10^-3^ after induction were classified as high risk.

### Statistical Analysis

Comparison of baseline clinical variables with gene expression was done using the non-parametric Mann-Whitney test. Categorical variables were compared by Fisher’s exact test. A p-value ≤ 0.05 (two-sided) was considered significant. Patients analyzed for *EMP1, CASP1*, and *NLRP3* expression were divided into two groups: low and high. This classification was done based on optimal cutoff determined using maximally selected rank statistics (maxstat) function for continuous variables, as provided in the “survminer” package. A widely cited web-based tool “KM-plotter” was used to perform the survival analysis for our custom data (https://kmplot.com/) (24). Therefore for each survival analysis, patients were now dichotomized into high expression and low expression groups of the respective gene. Furthermore, the same groups were utilized in the cox univariate and multivariate hazard model to analyze whether these associations are independent of other clinical variables.

Achievement of complete remission (CR) was assessed after completion of induction chemotherapy and presence of absolute neutrophil count >1000/µl, platelet count >100,000/µl, and hemoglobin >10 gm/dl, no PB blasts, less than 5% BM blasts, and absence of extramedullary leukemia. Relapse was defined as the re-emergence of blasts in the PB, more than 5% BM blasts, or the development of extramedullary leukemia.

EFS was defined as the time from diagnosis to the date of the last follow-up or the first event (i.e., induction failure, relapse, or death) (25). OS was defined as the time from diagnosis to death or the last follow-up. Patients lost to follow-up were censored at the last contact (25). RFS was defined as the time from the attainment of CR to the time of relapse or death or the last follow up. The last follow-up was carried out in April 2020. Estimated EFS, RFS, and OS probability were calculated by the Kaplan-Meier method, with the differences compared using a two-sided log-rank test. The relation between variables affecting EFS, RFS, and OS was evaluated by constructing multivariate Cox proportional hazard models. Covariates included in the full model of OS, RFS, and EFS were sex, TLC (≤50X10^9^/L, ≥50X10^9^/L), and age (<10 years vs. ≥10 years), gene expression, cytogenetics, BM remission status, and presence of MRD after the end of induction chemotherapy. All analyses were performed using the SPSS statistical software package, version 20.0/STATA software, version 11.

## Results

### Patient Characteristics

The median age of patients was 6 years (range 1–17 years). There were 97 males and 51 females. The median hemoglobin was 6.7 g/dl (range 2.1-14.2g/dl), median total leucocyte count (TLC) 14.8X10^9^/L (range 0.6–1021.4 X10^9^/L), and median platelet counts 42 X10^9^/L (range 2.6–500 X10^9^/L). The cytogenetic analysis revealed hyperdiploidy in 16 (10.8%), *BCR-ABL1* in 7 (4.72%), *ETV6-RUNX1* in 10 (6.75%), *E2A-PBX1* in 7 (4.72%), hypodiploidy in 3 (2.02%), other cytogenetic abnormalities in 2 (2.02%), negative in 91 (61.48%) and not evaluable in 12 (8.11%) patients. Sixty-four patients were categorized into NCI standard risk, while 84 patients into NCI high risk. Of total of 148 patients, three patients died during induction therapy. Out of the remaining 145 patients, 137 (94.48%) underwent remission. Post induction MRD was evaluated in 145 patients out of which 27 (18.62%) were MRD positive, while 3 patients were inevaluable for MRD. There were 28 (18.92%) PPRs.

### Association of Prednisolone Response With Clinicopathological Features

To determine the association of patient characteristics with prednisolone response in our patient cohort, we divided patients into PGR and PPR, based on day 8 blast count. Results from Chi-square test/Fisher exact test (**Table 1**) revealed that prednisolone response was not associated with age (p=0.82), sex (p=0.13), TLC at diagnosis (p=0.67), NCI risk (p=0.41), cytogenetics (p=0.68), post-induction MRD (p=0.81), or BM remission (p=0.15).

**Table 1:** Patient characteristics according to prednisolone response in B-ALL.

Baseline characteristics	Prednisolone good responders n (%)	Prednisolone poor responders n (%)	*P* value
Number of patients	120	28	
Age at diagnosis (in years)			0.82
1–9	85 (70.8)	21 (75)	
≥10	35 (29.2)	7 (25)	
Sex			0.13
Male	75 (62.5)	22 (78.6)	
Female	45 (37.5)	6 (21.4)	
TLC at diagnosis (X10^9^/L)			0.067
<50	90 (75)	16 (57.1)	
≥50	30 (25)	12 (42.9)	
NCI risk			0.41
Standard risk	54 (45)	10 (35.7)	
High risk	66 (55)	18 (64.3)	
Cytogenetics			0.68
BCR-ABL1	6 (5)	1 (3.6)	
E2A-PBX1	6 (5)	1 (3.6)	
ETV6-RUNX1	9 (7.5)	1 (3.6)	
MLL-rearranged	0	0	
Hyperdiploid	14 (11.7)	2 (7.1)	
Hypodiploid	3 (2.5)	0	
B-others	71 (59.2)	22 (78.6)	
Not available	11 (9.2)	1 (3.6)	
BM remission			0.15
Yes	114 (95.8)	23 (88.5)	
No	5 (4.2)	3 (11.5)	
Post-induction MRD			0.62
Negative	93 (78.2)	23 (84.6)	
Positive	23 (19.3)	4 (15.4)	
Inevaluable	3 (2.5)	0	

### Association of Gene Expression With Prednisolone Sensitivity

**Figure 1:**
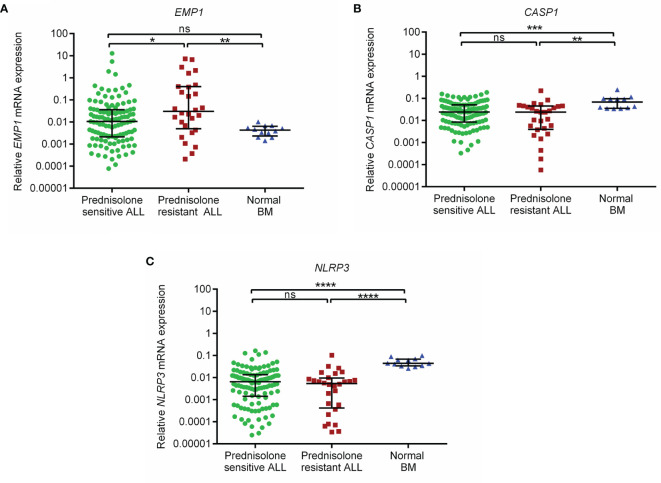
Relative expression of **(A)** *EMP1* **(B)** *CASP1*, and **(C)** *NLRP3* in prednisolone sensitive, resistant B-ALL patients and normal BM controls in the study cohort. Normal BM, normal bone marrow; ****p < 0.0001; ***p < 0.001; **p < 0.01; *p < 0.05; ns, not significant (p > 0.05).

**Figure 2:**
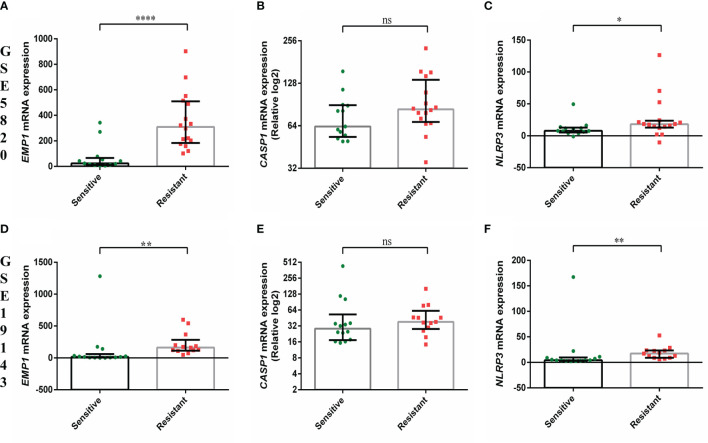
Comparison of gene expression of *EMP1*, *CASP1*, and *NLRP3* between prednisolone sensitive and resistance groups from **(A–C)** GSE5820 and **(D–F)** GSE19143 dataset. ****p < 0.0001; ***p < 0.001; **p < 0.01; *p < 0.05; ns, not significant (p > 0.05).

*EMP1* levels were higher in PPRs as compared to PGRs (p=0.015, **Figure 1A**). Its expression was higher in ALL patients as compared to normal BM controls (all cases versus controls p=0.045, **Supplementary Figure 1A**; PPRs versus controls p=0.0041 and PGRs versus controls p=0.093, **Figure 1A**). We did not find any association between *CASP1* and *NLRP3* expression with PRD sensitivity (p=0.25 and 0.24, respectively) (**Figures 1B, C**). CASP1 and *NLPR3* levels were lower in ALL patients compared to controls (*CASP1*: all cases versus controls p=0.0002, **Supplementary Figure 1B**; PPRs versus controls p=0.0017 and PGRs versus controls p=0.0003, **Figure 1B** and *NLRP3* all cases versus controls p<0.0001, **Supplementary Figure 1C**; PPRs versus controls p<0.0001 and PGRs versus controls p<0.0001, **Figure 1C**). We further determined the association of gene expression with prednisolone sensitivity in GSE5820 and GSE19143 datasets. This revealed that the mRNA expression of *EMP1* and *NLRP3* genes was higher in GC-resistant compared to GC-sensitive patients in both datasets (*EMP1*: GSE5820 p<0.0001, GSE19143 p=0.001, **Figures 2A, D**; *NLRP3*: p=0.022, GSE19143 p =0.002, **Figures 2C, F**). We did not find any difference between GC-resistant and GC-sensitive patients in both datasets with respect to expression of *CASP1* (GSE5820 p=0.17; GSE19143 p =0.18, **Figures 2B, E**).

### Association of Gene Expression With Patient Characteristics and Response to Induction Therapy

**Figure 3:**
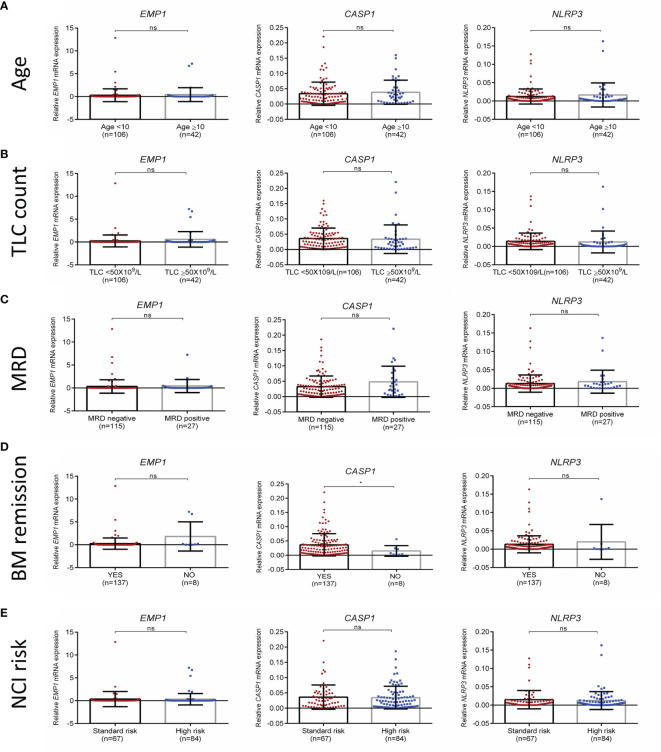
Association of *EMP1*, *CASP1*, and *NLRP3* expression with **(A)** age, **(B)** total leukocyte count at diagnosis **(C)** MRD status **(D)** BM remission status, and **(E)** NCI risk. TLC, total leukocyte count; NCI, national cancer institute, USA based risk stratification; BM, bone marrow; ****p < 0.0001; ***p < 0.001; **p < 0.01; *p < 0.05; ns, not significant (p > 0.05).

**Figure 4:**
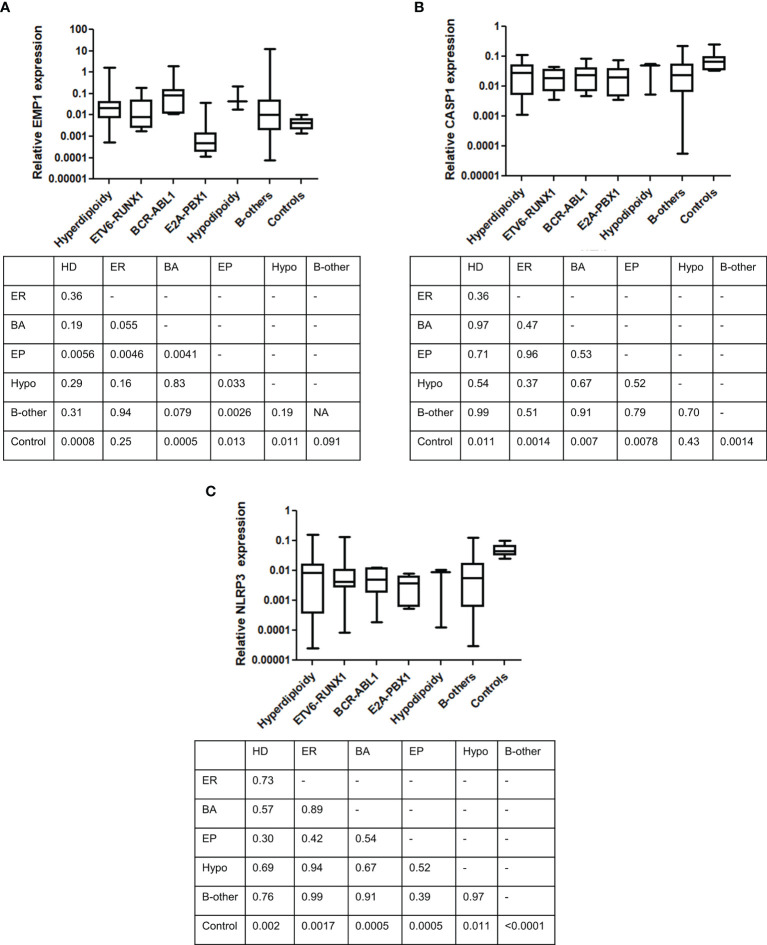
Comparison of gene expression between different cytogenetic subgroups. **(A)** *EMP1*, **(B)** *CASP1*, and **(C)** *NLRP3*. Table below each figure panel represents results of Mann-Whitney U test analysis between each respective group. ER, *ETV6-RUNX1*; EP, *E2A-PBX1*; BA, *BCR-ABL1*; Hypo, hypodiploidy; HD, hyperdiploidy.

We compared the expression of *EMP1*, *CASP1*, and *NLRP3* based on clinicopathological features. No association of expression of these genes was observed with age (**Figure 3A**), TLC count (**Figure 3B**), MRD (**Figure 3C**), and NCI risk (**Figure 3E**). Interestingly, among the three analyzed genes, higher expression of *CASP1* was associated with BM remission status (p=0.0487, **Figure 3D**), while *EMP1* and *NLRP3* did not exhibit any association. Further, none of these three genes exhibited a difference in expression based on sex (**Supplementary Figure 2A**) and CSF status (**Supplementary Figure 2B**). We also compared the expression of these genes among different cytogenetic subgroups (**Figure 4**). This revealed that *EMP1* had an association with cytogenetics (**Figure 4A**). The median *EMP1* mRNA level was lower in patients with *E2A-PBX1* as compared to other cytogenetic subtypes of B-ALL (*BCR-ABL1* p=0.004; hyperdiploidy p=0.0056; *ETV6-RUNX1* p=0.0046; hypodiploidy p=0.033 and B-others p=0.0026) and controls (p=0.013). *BCR-ABL1*, hyperdiploid and hypodiploid B-ALL had higher expression of *EMP1* compared to controls (p=0.0005, 0.0008, and 0.011, respectively). *CASP1* and *NLRP3* did not have any association with cytogenetics in our cohort (**Figures 4B, C**). All cytogenetic subtypes of B-ALL had lower expression of *CASP1* (except hypodiploid) and *NLRP3* compared to controls. We also utilized GEP data of ALL samples from the MILE study to compare the expression of these genes in ALL cytogenetic subtypes (**Supplementary Figure 3**). In the MILE dataset, *EMP1* expression was lower in E2A-PBX1 and ETV6-RUNX1 subtypes compared to other cytogenetic subtypes, while the BCR-ABL subtype showed the highest *EMP1* expression (**Supplementary Figure 3A**). Further, all ALL subtypes exhibited lower expression of *CASP1* and *NLRP3* (except MLL-R) compared to normal bone marrow (**Supplementary Figures 3B, C**, respectively), while *EMP1* expression was higher in all cytogenetic subtypes compared to normal bone marrow (**Supplementary Figure 3A**).

### *EMP1* Associated Pathways in B-ALL

**Figure 5:**
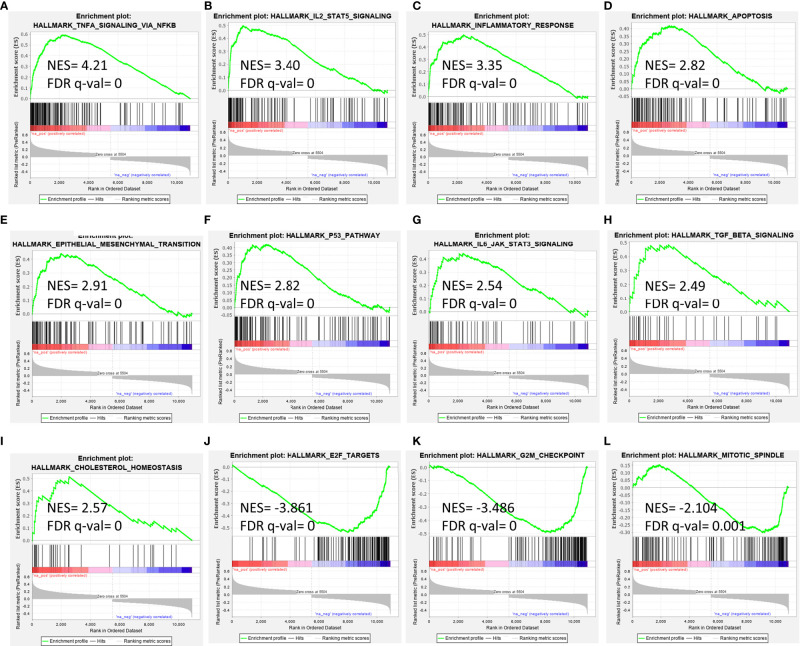
Gene set enrichment analysis for *EMP1* correlated genes in B-ALL **(A–I)** positively correlated pathways, **(J–L)** negatively correlated pathways. Gene expression data from diagnostic bone marrow samples of B-ALL from Leukemia MILE study was utilized to extract *EMP1* associated genes and analyzed by GSEA tool. NES, normalized enrichment score; FDR, false detection rate.

As *EMP1* exhibited overexpression in leukemic samples compared to normal bone marrow and also exhibited higher expression in PPR group compared to PGRs, we performed pathway analysis of *EMP1* correlated genes using B-ALL patients from MILE study dataset (**Figure 5**). As this dataset consists of both adult and pediatric cases, we corroborated the results with two more datasets (GSE5820 and GSE19143) which have only pediatric patients. We observed that *EMP1* expression was positively associated with TNF-alpha, IL-2-STAT5 signaling, inflammatory responses, and hypoxia in all three datasets (**Supplementary Table 1**). Further, other positively correlated pathways were also common in 2/3 datasets, such as IL6 Jak-Stat3 signaling, complement, TGF-beta signaling, androgen response, upregulated genes in KRAS signaling, p53 pathway. Negatively correlated genes were enriched in E2F targets, G2M checkpoint, and mitotic spindle formation in MILE dataset, while E2F targets was observed as negatively associated target in both MILE and GSE5820 dataset. Details of the pathways of all the datasets are given in **Supplementary Table 1**.

### Survival Analysis

**Figure 6:**
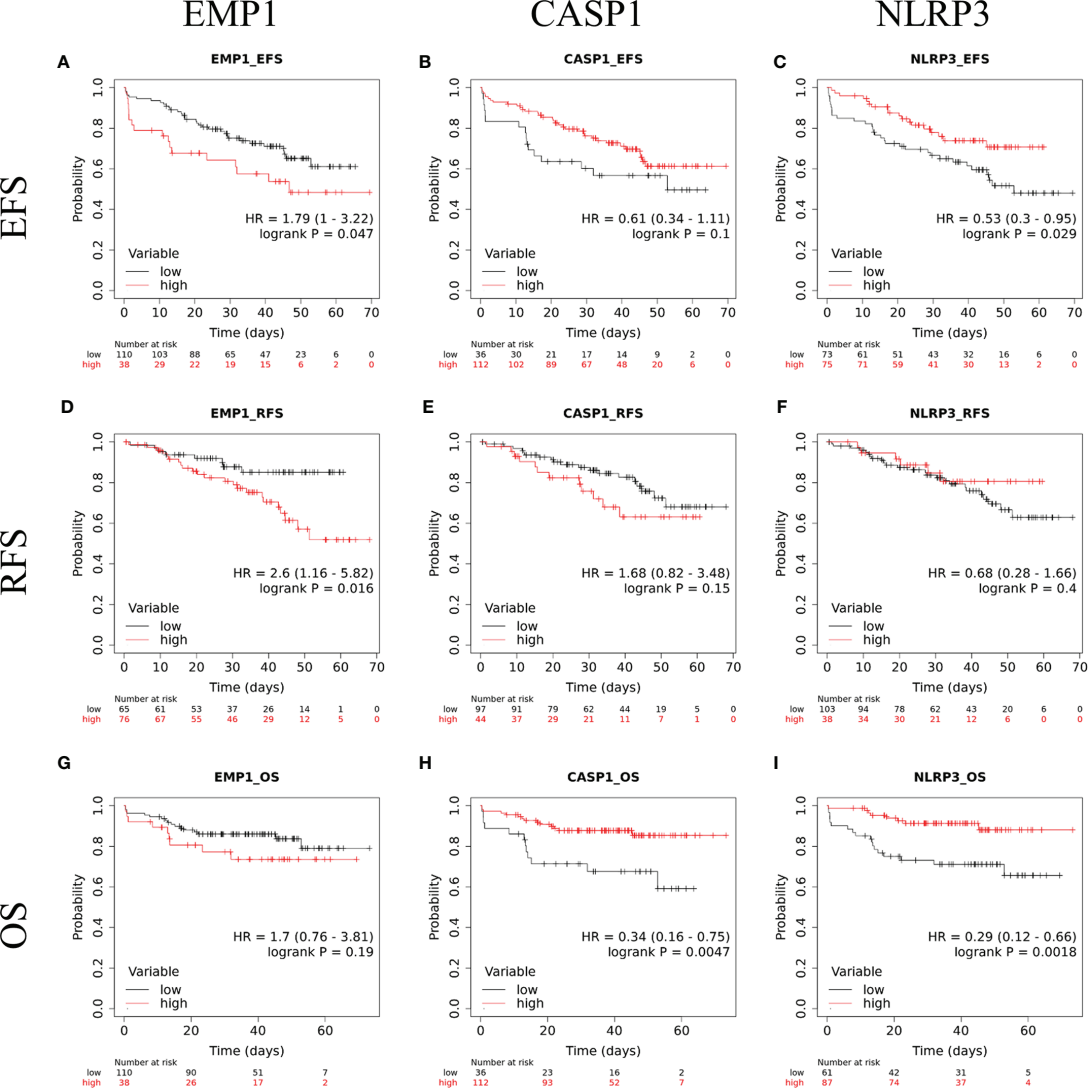
Kaplan Meir survival plots comparing EFS in B-ALL patients with **(A)** *EMP1*-high and *EMP1*-low expression **(B)** *CASP1*-high and *CASP1*-low expression **(C)** *NLRP3*-high and *NLRP3*-low expression; RFS in B-ALL patients with **(D)** *EMP1*-high and *EMP1*-low expression **(E)** *CASP1*-high and *CASP1*-low expression **(F)** *NLRP3*-high and *NLRP3*-low expression; OS in B-ALL patients with **(G)** *EMP1*-high and *EMP1*-low expression **(H)** *CASP1*-high and *CASP1*-low expression **(I)** *NLRP3*-high and *NLRP3*-low expression. HR, hazard ratio; EFS, event free survival; RFS, relapse free survival; OS, overall survival.

The follow up of the patients ranged from 0.5 to 73 months (median 37 months). Patients with high *EMP1* expression or low *NLRP3* had a worse 5-year EFS probability compared to patients with low *EMP1* expression (HR 1.79, 95% confidence interval 1 to 3.22, p=0.047, **Figure 6A**) or low *NLRP3* levels (HR 0.53, 95% confidence interval 0.3 to 0.95, p=0.029, respectively, **Figure 6C**), respectively. We did not find any association between *CASP1* expression with EFS (HR 0.61, 95% confidence interval 0.34 to 1.11, p=0.1), **Figure 6B**). Expression of *EMP1* was associated with poor RFS (HR 2.6, 95% confidence interval 1.16 to 5.82, p=0.016, **Figure 6D**), while *CASP1* and *NLRP3* did not exhibit any association with RFS (*CASP1*: HR 1.68, 95% confidence interval 0.82 to 3.48, p=0.15, **Figure 6E**; NLRP3: HR 0.68, 95% confidence interval 0.28 to 1.66, p=0.4, **Figure 6F**). We found low expression of *CASP1* and *NLRP3* to be associated with inferior OS (*CASP1*: high HR 0.34, 95% confidence interval 0.16–0.75, p=0.0047, **Figure 6H**; *NLRP3*: HR 0.29, 95% confidence interval 0.12–0.66, p=0.0018, **Figure 6I**). We did not find any association between *EMP1* expression and OS (HR 1.7, 95% confidence interval 0.76–3.81, p=0.19, **Figure 6G**). We also determined the prognostic associations of clinical and molecular features such as age, gender, WBC count, cytogenetic classification, MRD status, infiltration of cerebrospinal fluid (CSF), NCI risk, and prednisolone response status in the current patient cohort, shown in **Table 2**. To evaluate the independent prognostic value of *EMP1*, *CASP1*, and *NLRP3*, we performed multivariate Cox regression analysis. Among these genes, *EMP1* expression was not significantly associated with patient survival in multivariate analysis (**Table 3**). Higher *CASP1* expression was associated with better OS (HR 0.425, 95% confidence interval 0.182–0.990, p=0.048, **Table 4**), while higher *NLRP3* expression was associated with better EFS (HR 0.439, 95% confidence interval 0.236–0.818, p=0.010, **Table 5**) and OS (HR 0.310, 95% confidence interval 0.126–0.766, p=0.011, **Table 5**).

**Table 2:** Univariate analysis for prognostic association of target genes and other covariates in B-ALL patients.

Variables	Relapse free survival			Event free survival			Overall survival		
	HR	95% CI	P-value	HR	95% CI	P-value	HR	95% CI	P-value
**Age at diagnosis**									
<10 years	**Ref**			**Ref**			**Ref**		
≥10 years	**2.22**	**1.080–4.599**	**0.029**	**2.481**	**1.412–4.360**	**0.002**	**3.038**	**1.405–6.565**	**0.005**
**Sex**									
Male	Ref			Ref			Ref		
Female	1.361	0.640–2.896	0.424	1.158	0.644–2.083	0.622	0.898	0.407–1.980	0.790
**WBC count (X10^9^/L)**									
<50	Ref			Ref			Ref		
≥50	1.060	0.477–2.386	0.874	1.378	0.760–2.499	0.290	2.034	0.934–4.432	0.074
**Cytogenetics**									
ETV6-RUNX1/Hyperdiploidy	Ref			Ref			Ref		
BCR-ABL/E2A-PBX1/Hypodiploidy	3.144	1.026–9.636	0.045	2.219	0.917–5.365	0.077	2.051	0.550–7.647	0.284
B-others	0.841	0.308–2.292	0.736	0.825	0.390–1.746	0.617	1.141	0.383–3.400	0.812
**Minimal residual disease (MRD)**									
Negative	Ref			Ref			Ref		
Positive	**2.67**	**1.474–4.836**	**0.001**	**2.162**	**1.346–3.473**	**0.001**	1.461	0.700–3.046	0.312
**Cerebrospinal fluid**									
Negative	Ref			Ref			Ref		
Positive	1.713	0.232–12.629	0.597	**3.619**	**1.296–10.099**	**0.014**	**5.200**	**1.550–17.44**	**0.008**
**NCI risk**									
Standard risk	Ref			Ref			Ref		
High risk	1.139	0.561–2.312	0.718	1.458	0.823–2.582	0.196	1.933	0.840–4.449	0.121
**Prednisolone response**									
Sensitive	Ref			Ref		Ref			
Resistant	0.630	0.191–2.077	0.448	1.157	0.562–2.382	0.692	1.894	0.794–4.513	0.149
***EMP1* expression**									
Low	Ref			Ref			Ref		
High	**2.603**	**1.163–5.823**	**0.020**	**1.794**	**0.998–3.225**	**0.050**	1.698	0.756–3.813	0.199
***CASP1* expression**									
Low	Ref			Ref			Ref		
High	1.684	0.816–3.475	0.158	0.612	0.337–1.111	0.107	**0.344**	**0.159–0.747**	**0.007**
***NLRP3* expression**									
Low	Ref			Ref			Ref		
High	0.682	0.279–1.664	0.401	**0.530**	**0.297–0.945**	**0.032**	**0.287**	**0.124–0.663**	**0.003**

Bold values highlights significant associations.

**Table 3:** Multivariate Cox regression analysis for prognostic association of EMP1 in B-ALL patients.

Variables	Relapse free survival			Event free survival			Overall survival		
	HR	95% CI	P-value	HR	95% CI	P-value	HR	95% CI	P-value
**Age at diagnosis**									
<10 years	**Ref**			**Ref**			**Ref**		
≥10 years	**3.415**	**1.363–8.554**	**0.009**	**3.592**	**1.739–7.420**	**0.001**	**3.074**	**1.158–8.159**	**0.024**
**Sex**									
Male	Ref			Ref			Ref		
Female	2.244	0.907–5.550	0.080	1.157	0.592–2.259	0.669	0.651	0.270–1.570	0.340
**WBC count (X10^9^/L)**									
<50	Ref			Ref			Ref		
≥50	1.520	0.563–4.105	0.408	1.920	0.928–3.970	0.078	2.274	0.859–6.015	0.098
**Cytogenetics**									
ETV6-RUNX1/Hyperdiploidy	Ref			Ref			Ref		
BCR-ABL/E2A-PBX1/Hypodiploidy	6.242	1.611–24.176	0.008	3.076	1.097–8.620	0.033	2.017	0.474–8.579	0.342
B-others	1.119	0.363–3.449	0.844	0.870	0.374–2.020	0.746	0.888	0.268–2.942	0.846
**Minimal residual disease (MRD)**									
Negative	**Ref**			**Ref**			Ref		
Positive	**3.142**	**1.581–6.241**	**0.001**	**2.186**	**1.308–3.654**	**0.003**	1.386	0.649-2.960	0.398
**Cerebrospinal fluid**									
Negative	Ref			**Ref**			**Ref**		
Positive	0.952	0.100–9.001	0.966	**3.407**	**1.087–10.681**	**0.035**	**5.859**	**1.540–22.285**	**0.009**
**NCI risk**									
Standard risk	Ref			Ref			Ref		
High risk	0.430	0.150–1.230	0.116	0.623	0.282–1.375	0.242	0.777	0.246–2.447	0.667
**Prednisolone response**									
Sensitive	Ref			Ref			Ref		
Resistant	0.539	0.151–1.921	0.341	1.390	0.639–3.025	0.405	1.895	0.719–4.995	0.196
***EMP1* expression**									
Low	Ref			Ref			Ref		
High	2.114	0.916–4.880	0.079	1.214	0.622–2.368	0.569	1.035	0.411–2.609	0.940

Bold values highlights significant associations.

**Table 4:** Multivariate Cox regression analysis for prognostic association of CASP1 in B-ALL patients.

Variables	Relapse free survival			Event free survival			Overall survival		
	HR	95% CI	P-value	HR	95% CI	P-value	HR	95% CI	P-value
**Age at diagnosis**									
<10 years	**Ref**			**Ref**			**Ref**		
≥10 years	**3.502**	**1.392–8.808**	**0.008**	**3.683**	**1.811–7.490**	**0.000**	**3.037**	**1.174–7.858**	**0.022**
**Sex**									
Male	Ref			Ref			Ref		
Female	1.946	0.760–4.981	0.165	1.295	0.643–2.611	0.468	0.775	0.306–1.965	0.593
**WBC count (X10^9^/L)**									
<50	Ref			Ref			Ref		
≥50	1.710	0.628–4.653	0.293	1.779	0.866–3.656	0.117	1.924	0.733–5.049	0.183
**Cytogenetics**									
ETV6-RUNX1/Hyperdiploidy	Ref			Ref			Ref		
BCR-ABL/E2A-PBX1/Hypodiploidy	5.881	1.452–23.823	0.013	**3.243**	**1.160–9.064**	**0.025**	1.977	0.467–8.374	0.354
B-others	0.976	0.298–3.197	0.969	0.917	0.394–2.131	0.841	0.920	0.280–3.023	0.891
**Minimal residual disease (MRD)**									
Negative	**Ref**			**Ref**			Ref		
Positive	**3.312**	**1.681–6.527**	**0.001**	**2.193**	**1.332–3.608**	**0.002**	1.249	0.597–2.612	0.554
**Cerebrospinal fluid**									
Negative	Ref			**Ref**			**Ref**		
Positive	1.054	0.100–11.098	0.964	**3.472**	**1.118–10.780**	**0.031**	**5.910**	**1.557–22.438**	**0.009**
**NCI risk**									
Standard risk	Ref			Ref			Ref		
High risk	0.440	0.160–1.211	0.112	0.612	0.280–1.336	0.218	0.767	0.242–2.425	0.652
**Prednisolone response**									
Sensitive	Ref			Ref			Ref		
Resistant	0.654	0.186–2.296	0.508	1.410	0.658–3.021	0.376	1.579	0.600–4.156	0.355
***CASP1* expression**									
Low	Ref			Ref			**Ref**		
High	1.268	0.548–2.933	0.578	0.622	0.323–1.198	0.156	**0.425**	**0.182–0.990**	**0.048**

Bold values highlights significant associations.

**Table 5:** Multivariate Cox regression analysis for prognostic association of NLRP3 in B-ALL patients.

Variables	Relapse free survival			Event free survival			Overall survival		
	HR	95% CI	P-value	HR	95% CI	P-value	HR	95% CI	P-value
**Age at diagnosis**									
<10 years	**Ref**			**Ref**			**Ref**		
≥10 years	**3.866**	**1.547–9.657**	**0.004**	**3.904**	**1.900–8.021**	**0.000**	**3.192**	**1.187–8.581**	**0.021**
**Sex**									
Male	Ref			Ref			Ref		
Female	2.106	0.853–5.194	0.106	1.293	0.651–2.565	0.462	0.731	0.297–1.799	0.495
**WBC count (X10^9^/L)**									
<50	Ref			Ref			Ref		
≥50	1.574	0.588–4.212	0.366	1.817	0.894–3.691	0.099	1.512	0.559–4.088	0.415
**Cytogenetics**									
ETV6-RUNX1/Hyperdiploidy	Ref			Ref			Ref		
BCR-ABL/E2A-PBX1/Hypodiploidy	**6.122**	**1.558–24.046**	**0.009**	3.224	1.145–9.078	0.027	1.721	0.395–7.497	0.469
B-others	1.112	0.354–3.486	0.855	0.809	0.350–1.872	0.622	0.901	0.274–2.957	0.864
**Minimal residual disease (MRD)**									
Negative	**Ref**			**Ref**			Ref		
Positive	**3.393**	**1.720–6.696**	**0.000**	**2.288**	**1.395–3.753**	**0.001**	1.358	0.649–2.840	0.416
**Cerebrospinal fluid**									
Negative	Ref			**Ref**			**Ref**	**1.607–**	
Positive	1.692	0.158–18.015	0.663	**4.169**	**1.336–13.014**	**0.014**	**6.236**	**24.186**	**0.008**
**NCI risk**									
Standard risk	Ref			Ref			Ref		
High risk	0.414	0.151–1.136	0.087	0.571	0.258–1.263	0.167	0.811	0.250–2.625	0.727
**Prednisolone response**									
Sensitive	Ref			Ref			Ref		
Resistant	0.613	0.174–2.150	0.445	1.464	0.684–3.133	0.326	1.802	0.711–4.565	0.214
***NLRP3* expression**									
Low	Ref			**Ref**			**Ref**		
High	0.688	0.247–1.914	0.475	**0.439**	**0.236–0.818**	**0.010**	**0.310**	**0.126–0.766**	**0.011**

Bold values highlights significant associations.

## Discussion

PRD resistance in pediatric ALL is one of the major causes of therapeutic failure (2, 6–8). Previous studies tried to elucidate the molecular mechanisms of PRD resistance in ALL by comparing microarray gene expression patterns in PRD sensitive and resistant leukemia cells (14–16). They identified upregulation of *EMP1* and NALP3 inflammasome pathway key components, *CASP1* and *NLRP3* in PRD resistant patients. More recently, an integrative genomic approach was utilized in an attempt to identify genomic and epigenomic determinants of drug resistance (12). Using this approach, several novel molecular features contributing to GC response in ALL were identified (12). It was further observed that *EMP1* mRNA, *CASP1* methylation, and mRNA and *NLRP3* methylation levels differ between GC resistant and sensitive patients. In the current study, we evaluated the association between the expression of these three genes in pediatric B-ALL and response to PRD and also assessed their impact on clinical outcome.

We found a positive association between response to PRD and *EMP1* expression. Our findings are similar to those of previously reported studies, which also observed a significant overexpression of *EMP1* gene in PRD resistant leukemic cells (12, 13, 16). Aries et al. also showed that knockdown of *EMP1* gene resulted in moderate sensitization of leukemic cells to PRD (16). We also found higher expression of *EMP1* gene in GC-resistant samples in GEP datasets – GSE5820 and GSE19143. Further, none of these genes were associated with clinicopathological features including age, sex, TLC at diagnosis, CSF involvement, MRD status, and NCI risk. We observed that higher expression of *CASP1* was associated with an attainment of BM remission, in agreement with a previous report (16). Further, we observed *EMP1* expression to be higher in *BCR-ABL1*-positive B-ALL cases compared to controls. To validate these findings, we used the MILE dataset and compared *EMP1* expression in different cytogenetic subtypes (20). Consistent with our patient cohort, analysis of MILE data also confirmed higher expression of *EMP1* in the BCR-ABL subtype. *EMP1* is known to be one of several high confidence, conserved IKZF1-repressed genes (26). In B-ALL with IKZF1 alterations, *EMP1* is transcriptionally activated resulting in the proliferation of leukemic cells (26). This may be responsible for increased expression of *EMP1* in *BCR-ABL1*-positive B-ALL which frequently have IKZF1 deletions. Interestingly, we also observed that compared to other cytogenetic subtypes of B-ALL, *E2A-PBX1*-positive B-ALL exhibited the lowest expression of *EMP1*. It will be interesting to determine whether the subgroup-specific expression has any prognostic value in ALL. Aries et al., have previously compared *EMP1* expression between PRD resistant and sensitive patients within different cytogenetic subgroups (16). They identified that basal *EMP1* expression levels in prednisolone-sensitive B-ALL patients were highest in hyperdiploid patients>BCR-ABL1-like cases while no significant difference was observed between ETV6-RUNX1 and B-others. They did not show data of *EMP1* expression in E2A-PBX1 subtype.

EMP1, a small hydrophobic transmembrane glycoprotein, is implicated in the regulation of cell cycle and cell survival as silencing of *EMP1* results in cell-cycle arrest and apoptosis (16). It also has a role in the migration and adhesion of leukemic cells (27). Interaction with mesenchymal stem cells has been shown to induce GC resistance in leukemic cells. It has been demonstrated that EMP1 mediates PRD resistance induced by the interaction of leukemic cells with mesenchymal stem cells (16). EMP1 is upregulated in glioblastoma multiforme and its inhibition decreases proliferation and invasiveness of tumor (28–30). In contrast, in carcinoma of the nasopharynx, stomach, breast, urothelium, and prostate, EMP1 reduces cell migration and invasion and increases apoptosis and caspase-9 expression (27, 31–34). *EMP1* expression in non-small cell lung carcinoma is associated with clinical resistance to gefitinib (35).

On GSEA analysis for EMP1 associated molecular pathways in B-ALL, we observed that genes that are positively correlated with *EMP1* expression are enriched in oncogenic pathways such as TNF-alpha, IL-2-STAT5 signaling, inflammatory responses, and hypoxia. Kagoya et al. (2014) showed that in myeloid leukemia, NF-κB/TNF-α signaling is maintained which contributes to leukemia progression (35). It is hypothesized that there is potential crosstalk between glucocorticoid and NF-kB pathway which mediates breast cancer progression and survival (36). STAT5 is a transcription factor which plays a pivotal role in B cell transformation. STAT5 can be stimulated by IL2 secreted by immune cells. High STAT5 expression leads to more aggressive disease and mediates IL-2 induced glucocorticoid resistance (37). Inflammatory responses are dysregulated in ALL (38) and chronic inflammation in addition to genetic changes aids in the development of myeloid leukemia (39). Hypoxia is common in the bone-marrow environment. Hypoxia-inducible factor-1α (HIFA), a transcription factor induced during hypoxia has been shown to regulate multiple mechanisms for oncogenic adaptations in leukemia (40, 41) and is associated with poor clinical outcomes. Previous studies have shown that *EMP1* is indeed regulated by HIF-1α (42). Further studies are required to explore the association of *EMP1* expression and hypoxia in ALL. Moreover, pathway analysis showed *EMP1* is negatively correlated with cell cycle pathways such as E2F, mitotic spindle, and G2M checkpoints. A previous study on esophageal carcinoma reported overexpression of *EMP1* inhibits cell proliferation by cell cycle arrest at S phase (43), whereas in a leukemic cell line, EMP1 inhibition has been shown to induce cell cycle arrest at G1 phase (16).

High *EMP1* was associated with poor EFS in ALL in previous studies (16, 26). Witkowski et al. (2017) reported that high *EMP1* expression was associated with poor EFS in both IKZF1 deleted and non-deleted groups (26). Similar to previous studies, we found an association of EFS and RFS with *EMP1* expression levels. However, on multivariate analysis, we did not find a statistically significant association between EFS or RFS and *EMP1* levels. While this is in agreement with the previous studies where it has been demonstrated that initial response to prednisolone (measured by day 8 blast count) has limited prognostic utility compared to MRD (44), nevertheless, it is a widely used method to risk stratify the patients, in compliance with the BFM or other modified protocols based on BFM (23). Most importantly, as the high-risk patient identified by prednisolone response are given intensified therapy, the prognostic impact of PRD response cannot be directly compared to MRD in this case, as suggested by Conter et al. (44). While higher expression of *EMP1* was associated with prednisolone resistance as measured by day 8 blast count, none of the genes analyzed in this study was associated with MRD status. This might be due to the complexity of the disease mechanism, which may get modulated during the treatment. Interestingly, during induction therapy with prednisolone, day 14 blast count status, but not day 35 blast count status is correlated with day 8 blast count in BFM protocol (45).

In the study by Paugh et al. (2015), mRNA of *CASP1* and *NLRP3* were overexpressed at 1.6 fold and 2.4 fold, respectively, in GC-resistant ALL cells compared to GC-sensitive ALL cells (15, 46). Contrary to this, we did not observe any association of PRD response with *CASP1* gene in our patient cohort or GEP datasets GSE5820 and GSE19143, while expression of *NLRP3* was higher in GC resistant groups compared to GC sensitive groups in GEP dataset, but not in our patient cohort. This might be because the method they used to define PRD sensitivity was based on *in vitro* drug sensitivity assay, while we utilized clinical assessment data (initial response to PRD) as the comparative measure. This is also consistent with the previous study by Stam et al. (2010), from which GSE19143 data were extracted (19). In that study also, the authors determined differential genes based on *in vitro* drug sensitivity assay, where SLC16A1 (gene encoding MCT-1) was found to be overexpressed in GC resistant cells, but its expression was not correlated with *in vivo* prednisolone response (19). Among the three analyzed genes in our study, post-induction clinical remission was higher in patients with high *CASP1*. We also observed an association of higher *CASP1* expression with better OS. Additionally, higher expression of *NLRP3* was associated with better OS and EFS. This is contrary to the fact that transcriptional activation of CASP1 and NLRP3 expression by DNA methylation was previously shown to induce GC resistance in ALL. The prognostic associations have not been previously studied. Therefore, while these genes were not associated with PRD response in our study, the reason for association of higher expression of these genes with better patient prognosis requires further study in a larger cohort. Interestingly, analysis of our patient cohort and MILE study data revealed that both *CASP1* and *NLRP3* exhibited reduced expression in ALL samples compared to control BM.

Notably, we could not observe a significant association between cytogenetics with *CASP1* and *NLRP3* expression in our patient cohort, while in MILE study data, compared to all other cytogenetic subtypes, ETV6-RUNX1 exhibited the lowest expression of *CASP1* and *NLRP3*. Furthermore, the expression pattern of *CASP1* was similar to *EMP1* as its expression was higher in hyperdiploid and BCR-ABL subtypes. *NLRP3* expression was highest in the MLL-R subtype compared to other cytogenetic subtypes in the MILE dataset. The observed variations for expression of *CASP1* and *NLRP3* in our data compared to MILE study data might be due to the limited number of patients in our study. Further, some variation might have arisen due to the inclusion of adult ALL samples in the MILE study. Moreover, the possibility of ethnicity-based variations and treatment based variations in these results cannot be avoided, which may also contribute to the observation that response to PRD did not exhibit any association with patient prognosis.

Taken together, our study demonstrates the prognostic relevance of *EMP1*, *CASP1*, and *NLRP3* in pediatric B-ALL. A limitation of the study is the number of patients recruited. Nevertheless, these findings provide the foundation for future studies to identify potential drugs that can inhibit *EMP1* in B-ALL and other diseases where PRD is used as therapy. It would also be interesting to explore the detailed functional role of these proteins in other hematological malignancies.

## Data Availability Statement

The datasets presented in this study can be found in online repositories. The names of the repository/repositories and accession number(s) can be found in the article/**Supplementary Material**.

## Ethics Statement

The studies involving human participants were reviewed and approved by Institutional Ethics Committee, All India Institute of Medical Sciences, New Delhi, India. Written informed consent to participate in this study was provided by the participants’ legal guardian/next of kin.

## Author Contributions

RK and AC designed the study. JS, SK, MA, DV, MSA, and GS performed experiments, analyzed data, and contributed to writing the paper. JP, SC, and AS provided essential experimental tools and analyzed data. AR, PT, and AC helped in the diagnostic workup. SB and DP treated the patients and provided clinical details. All authors contributed to the article and approved the submitted version.

## Funding

This work is supported by the Wellcome Trust/DBT India Alliance Fellowship (grant number: IA/CPHI/17/1/503333) awarded to AC. Financial support from All India Institute of Medical Sciences, New Delhi, India as an intramural project (Grant number A487 to AC) is also acknowledged.

## Conflict of Interest

The authors declare that the research was conducted in the absence of any commercial or financial relationships that could be construed as a potential conflict of interest.
