# Supplementary material for: Prognostic Relevance of Expression of EMP1, CASP1, and NLRP3 Genes in Pediatric B-Lineage Acute Lymphoblastic Leukemia
Source: Front Oncol. 2021 Mar 5;11:606370. doi: 10.3389/fonc.2021.606370 (PMC7973229; doi:10.3389/fonc.2021.606370)
Supplement: Supplementary file 1 [file DataSheet_1.doc]

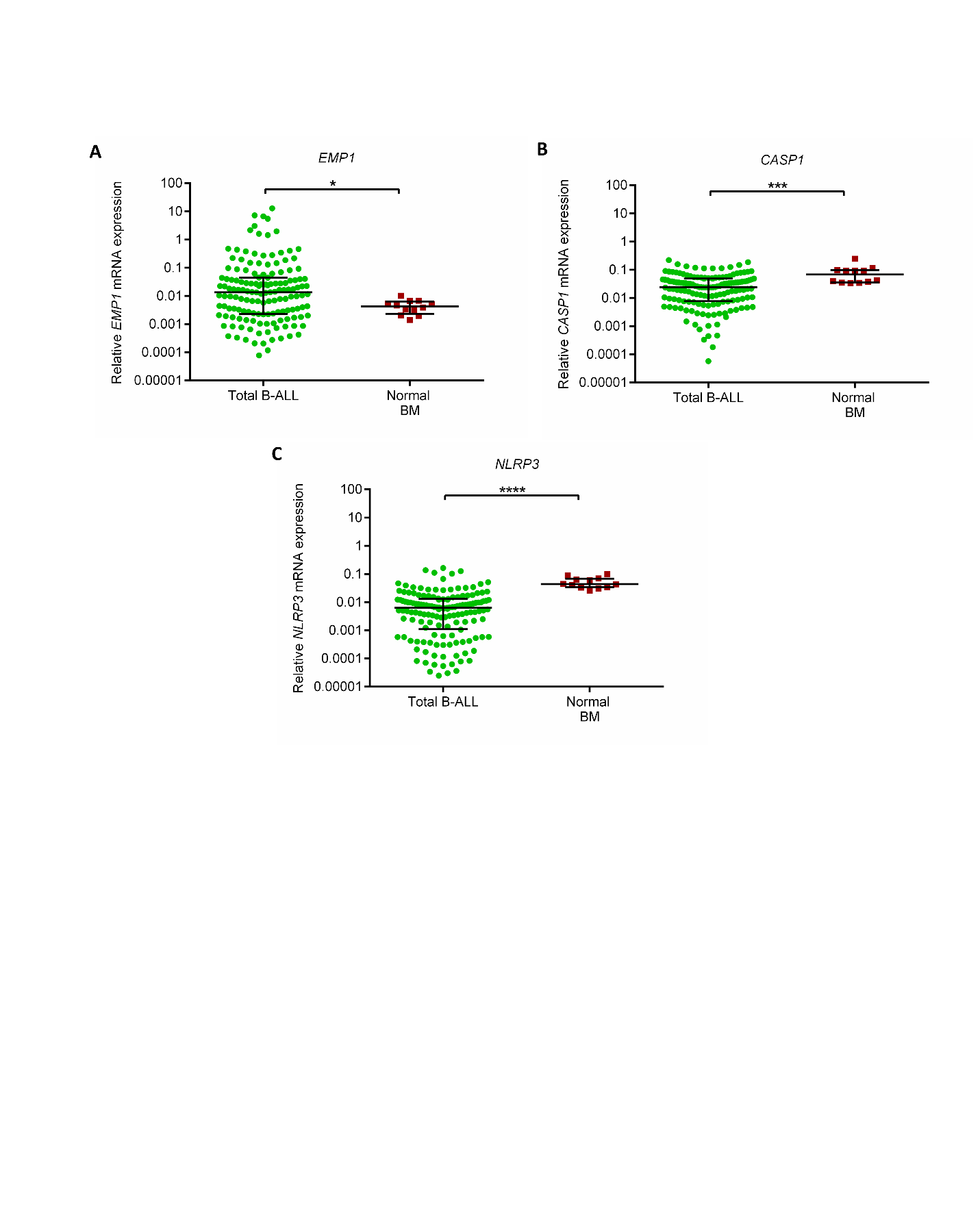


**Supplementary Figure 1**: Comparison of expression of target genes between B-ALL and normal bone marrows. (A) EMP1 (B) CASP1, and (C) NLRP3. ****p<0.0001; ***p<0.001; **p<0.01; *p<0.05; ns, not significant (p>0.05).


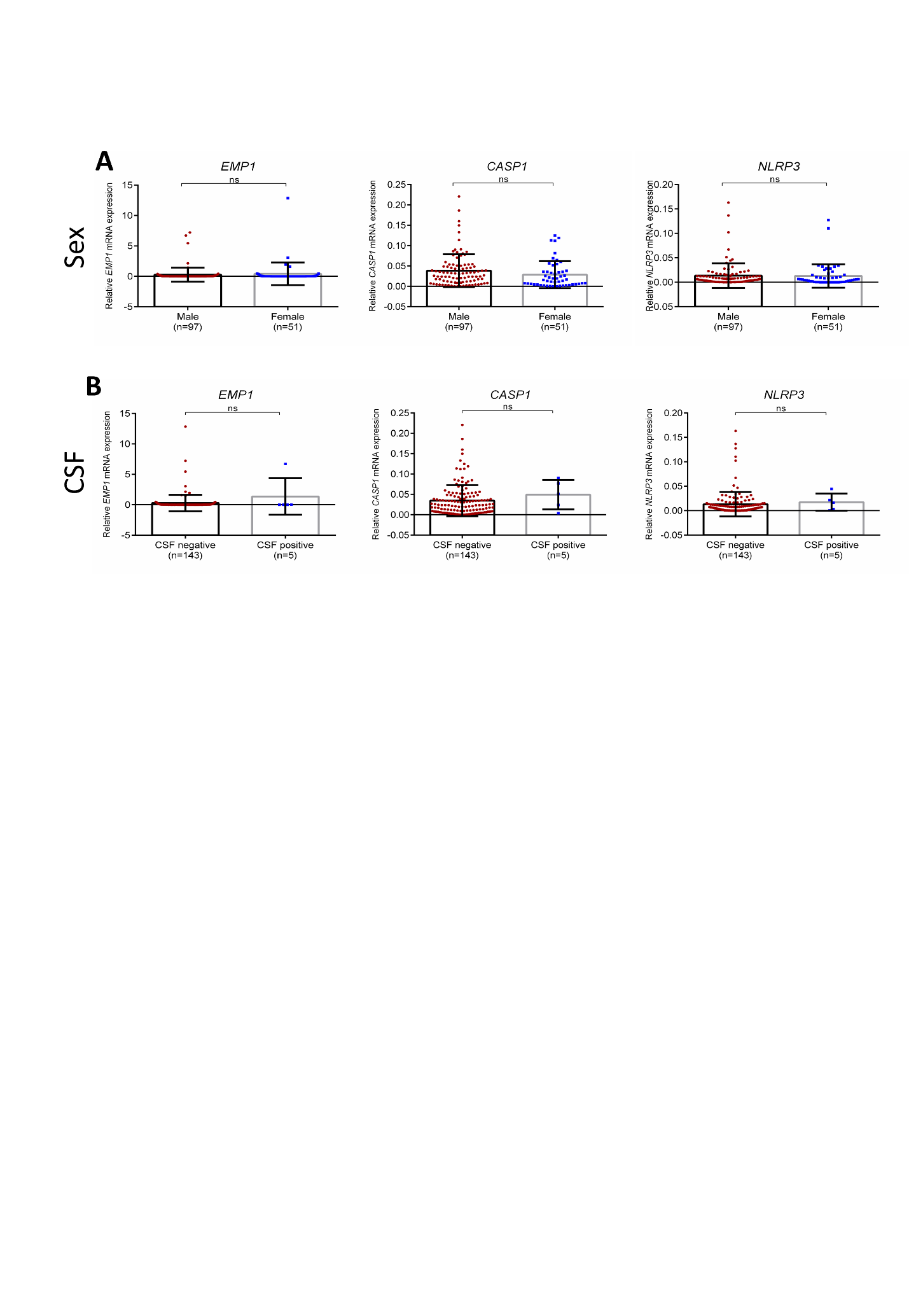
**Supplementary Figure 2**: Association of expression of EMP1, CASP1, and NLRP3 with (A) sex and (B) CSF status. CSF, cerebrospinal fluid; ****p<0.0001; ***p<0.001; **p<0.01; *p<0.05; ns, not significant (p>0.05).


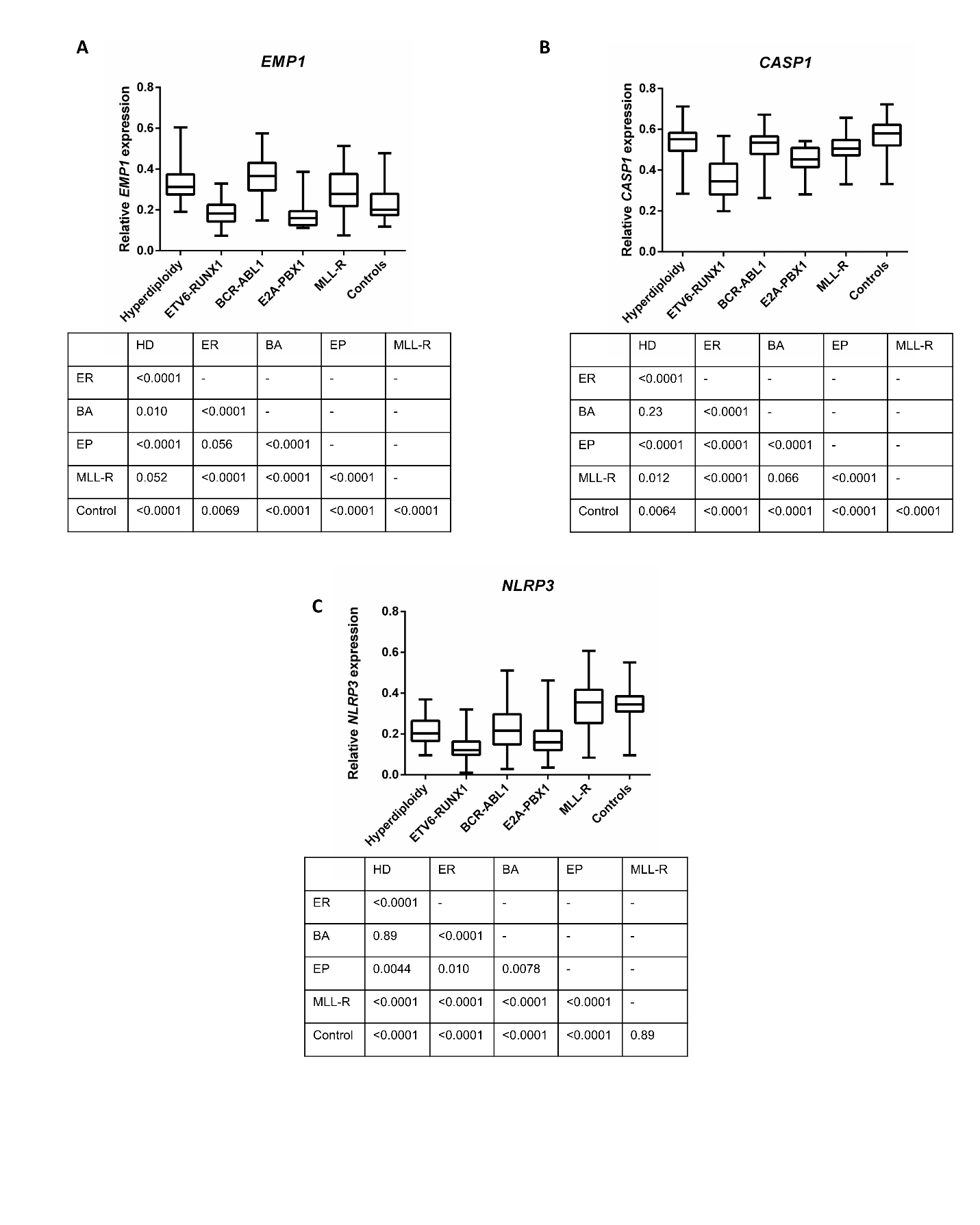
 **Supplementary Figure 3.** Comparison of expression of gene expression between different cytogenetic subgroups from MILE study dataset. (A) *EMP1*, (B) *CASP1,* and (C) *NLRP3.* Table below each figure panel represents results of Mann-Whitney U test analysis between each respective group. ER, *ETV6-RUNX1*; EP, *E2A-PBX1*; BA, *BCR-ABL1*; Hypo, hypodiploidy; HD, hyperdiploidy.

**Supplementary Table 1**: Gene set enrichment analysis for *EMP1* correlated genes in public datasets.

| **GSE5820** | | | **GSE19143** | | | **MILE** | | |
| --- | --- | --- | --- | --- | --- | --- | --- | --- |
| **Positively Correlated Pathways** | | | | | | | | |
| **NAME** | **NES** | **FDR q-value** | **NAME** | **NES** | **FDR q-value** | **NAME** | **NES** | **FDR q-value** |
| **TNFA SIGNALING VIA NFKB** | 5.571 | 0 | **TNFA SIGNALING VIA NFKB** | 3.290 | 0 | **TNFA SIGNALING VIA NFKB** | 4.211 | 0 |
| **INFLAMMATORY RESPONSE** | 3.487 | 0 | **INFLAMMATORY RESPONSE** | 2.174 | 0.015571 | **IL2 STAT5 SIGNALING** | 3.403 | 0 |
| **IL2 STAT5 SIGNALING** | 3.465 | 0 | **HYPOXIA** | 2.147 | 0.014538 | **INFLAMMATORY RESPONSE** | 3.350 | 0 |
| **APOPTOSIS** | 2.666 | 0 | **IL2 STAT5 SIGNALING** | 2.125 | 0.013151 | **HYPOXIA** | 2.982 | 0 |
| MTORC1 SIGNALING | 2.559 | 0 | **ANDROGEN RESPONSE** | 2.115 | 0.01052 | EPITHELIAL MESENCHYMAL TRANSITION | 2.908 | 0 |
| **IL6 JAK STAT3 SIGNALING** | 2.524 | 0.000258 |  |  |  | **P53 PATHWAY** | 2.823 | 0 |
| PROTEIN SECRETION | 2.224 | 0.002781 |  |  |  | **APOPTOSIS** | 2.817 | 0 |
| **COMPLEMENT** | 2.113 | 0.005484 |  |  |  | COAGULATION | 2.797 | 0 |
| UV RESPONSE DN | 2.112 | 0.004875 |  |  |  | CHOLESTEROL HOMEOSTASIS | 2.572 | 0 |
| **HYPOXIA** | 2.018 | 0.008381 |  |  |  | **IL6 JAK STAT3 SIGNALING** | 2.536 | 0 |
| **TGF BETA SIGNALING** | 2.005 | 0.008305 |  |  |  | **TGF BETA SIGNALING** | 2.486 | 0 |
| **ANDROGEN RESPONSE** | 1.971 | 0.010677 |  |  |  | ALLOGRAFT REJECTION | 2.465 | 0 |
| HEME METABOLISM | 1.909 | 0.014198 |  |  |  | ESTROGEN RESPONSE EARLY | 2.359 | 0 |
| **KRAS SIGNALING UP** | 1.885 | 0.015438 |  |  |  | **KRAS SIGNALING UP** | 2.334 | 0 |
| PI3K AKT MTOR SIGNALING | 1.838 | 0.020438 |  |  |  | INTERFERON GAMMA RESPONSE | 2.282 | 0 |
| **P53 PATHWAY** | 1.764 | 0.031606 |  |  |  | ANGIOGENESIS | 2.222 | 0.0000951 |
|  |  |  |  |  |  | APICAL JUNCTION | 2.098 | 0.000472 |
|  |  |  |  |  |  | MYOGENESIS | 2.065 | 0.001049947 |
|  |  |  |  |  |  | **COMPLEMENT** | 2.030 | 0.001155018 |
|  |  |  |  |  |  | **ANDROGEN RESPONSE** | 1.867 | 0.00467151 |
|  |  |  |  |  |  | ESTROGEN RESPONSE LATE | 1.666 | 0.018443352 |
|  |  |  |  |  |  | WNT BETA CATENIN SIGNALING | 1.639 | 0.021328835 |
| **Negatively Correlated Pathways** | | | | | | | | |
| E2F TARGETS | -2.174 | 0.016511 | **No observation** |  |  | E2F TARGETS | -3.861 | 0 |
|  |  |  |  |  |  | G2M CHECKPOINT | -3.485 | 0 |
|  |  |  |  |  |  | MITOTIC SPINDLE | -2.104 | 0.001306658 |

NES, normalized enrichment score; FDR-q value, false detection rate corrected p-value; UP, upregulated genes; DN, downregulated genes.
